# Bioluminescence imaging of a tumor-selective, thymidine kinase-defective vaccinia virus Guang9 strain after intratumoral or intraperitoneal administration in mice

**DOI:** 10.18632/oncotarget.20788

**Published:** 2017-09-08

**Authors:** Yuedi Ding, Jun Fan, Lili Deng, Ying Peng, Jue Zhang, Biao Huang

**Affiliations:** ^1^ Key Laboratory of Nuclear Medicine, Ministry of Health, Jiangsu Key Laboratory of Molecular Nuclear Medicine, Jiangsu Institute of Nuclear Medicine, Wuxi, Jiangsu, China

**Keywords:** bioluminescence imaging, vaccinia virus, Tian Tan strain Guang9, tumor selectivity

## Abstract

Vaccinia virus has been used as an oncolytic virus because of its capacity to preferentially infect tumors rather than normal tissues. The vaccinia Tian Tan strain, used as a vaccine against smallpox for millions of people in China, is a promising candidate for cancer therapy. In this study, we constructed an attenuated Tian Tan strain of Guang9 with a disrupted thymidine kinase gene to enhance tumor selectivity and an inserted firefly luciferase to monitor the viral distribution by *in vivo* bioluminescence imaging. Living animal imaging confirmed the high specificity of vaccinia Guang9 for tumor targeting after intratumoral and intraperitoneal administration. In addition, the vaccinia Guang9 strain produced higher *in vivo* luciferase activity and endured longer in immunocompromised nude mice than in immunocompetent C57BL/6 mice, all of which had been tumor-challenged. The luciferase activity and viral titers in excised tissues confirmed these conclusions. These data provide evidence for the safety and efficacy of the clinical application of vaccinia virus, which would be a promising approach for cancer therapy.

## INTRODUCTION

Despite advances in conventional therapies, we lack knowledge and effective treatments for many cancers [1]. The major treatment options for cancers, including surgery, radiation therapy, and chemotherapy, have been combined to improve outcomes for solid tumors [2, 3]. However, for patients who relapse after surgery or have unresectable disease, these options are only palliative treatments [4]. Thus, new cancer therapies with complementary mechanisms of action are needed.

Cancer therapy using oncolytic viruses represents a promising new approach for controlling tumors. The therapy is based on the capacity of oncolytic viruses to specifically target and lyse tumor cells to induce antitumor effects [5]. Herpes simplex virus (HSV) [6, 7] and adenovirus (Ad) [8], examples of oncolytic viruses studied to date, have been used in clinical trials. The vaccinia virus, popular for its role in the eradication of smallpox, has also been used as an oncolytic virus because of its ability to preferentially infect tumors rather than normal tissues [9–11]. In addition, vaccinia virus possesses a large linear double-stranded DNA genome consisting of approximately 250 genes, with the capacity for the insertion of therapeutic transgenes, such as the human granulocyte-macrophage colony-stimulating factor (hGM-CSF) gene [12–14].

The vaccinia virus Tian Tan strain (VTT), used as a vaccine against smallpox for millions of people in China before 1980, exhibits good immunogenicity, moderate reactogenicity, and relatively mild complications in humans. Moreover, to obtain a safer and more effective attenuated strain of vaccinia virus, the vaccinia virus Guang9 strain (VG9) was derived from VTT using a traditional singleplaque purification method [15, 16]. Therefore, vaccinia VG9 is a suitable strain for cancer therapy and could mediate anti-tumor effects as both an oncolytic agent and a viral vector for therapeutic gene delivery.

Vaccinia virus displays a natural tumor tropism and appears to selectively target tumors after systemic administration. In addition, genetic modification of the virus, such as the deletion of the gene edcoding thymidine kinase (TK), could improve its tumor specificity. The vaccinia virus without TK requires thymidine triphosphate from the nucleotide pool present in dividing cells for DNA synthesis. This leads to preferential viral replication in dividing cells and enhances the tumor selectivity of vaccinia virus [9, 13].

Many kinds of vaccinia virus have been constructed and tested in clinical trials, including Wyeth strain [13, 17], Western Reserve (WR) strain [14], Lister strain [18], and Modified Vaccinia Virus Ankara (MVA) [19]. However, research on VTT, which originates from China, has only recently started. The purpose of this study was to evaluate tumor specificity of VG9, the attenuated VTT strain with a disruption in the TK gene for tumor selectivity and the insertion of firefly luciferase (Luc) for bioluminescence imaging. After intratumoral or intraperitoneal administration, we monitored viral replication and distribution in real time using an *in vivo* bioluminescence imaging method in both immunocompetent and immunocompromised mice in 2 models of tumor challenge.

## RESULTS

### Vaccinia VG9-Luc is capable of infecting B16 cells *in vitro*

**Figure 1:**
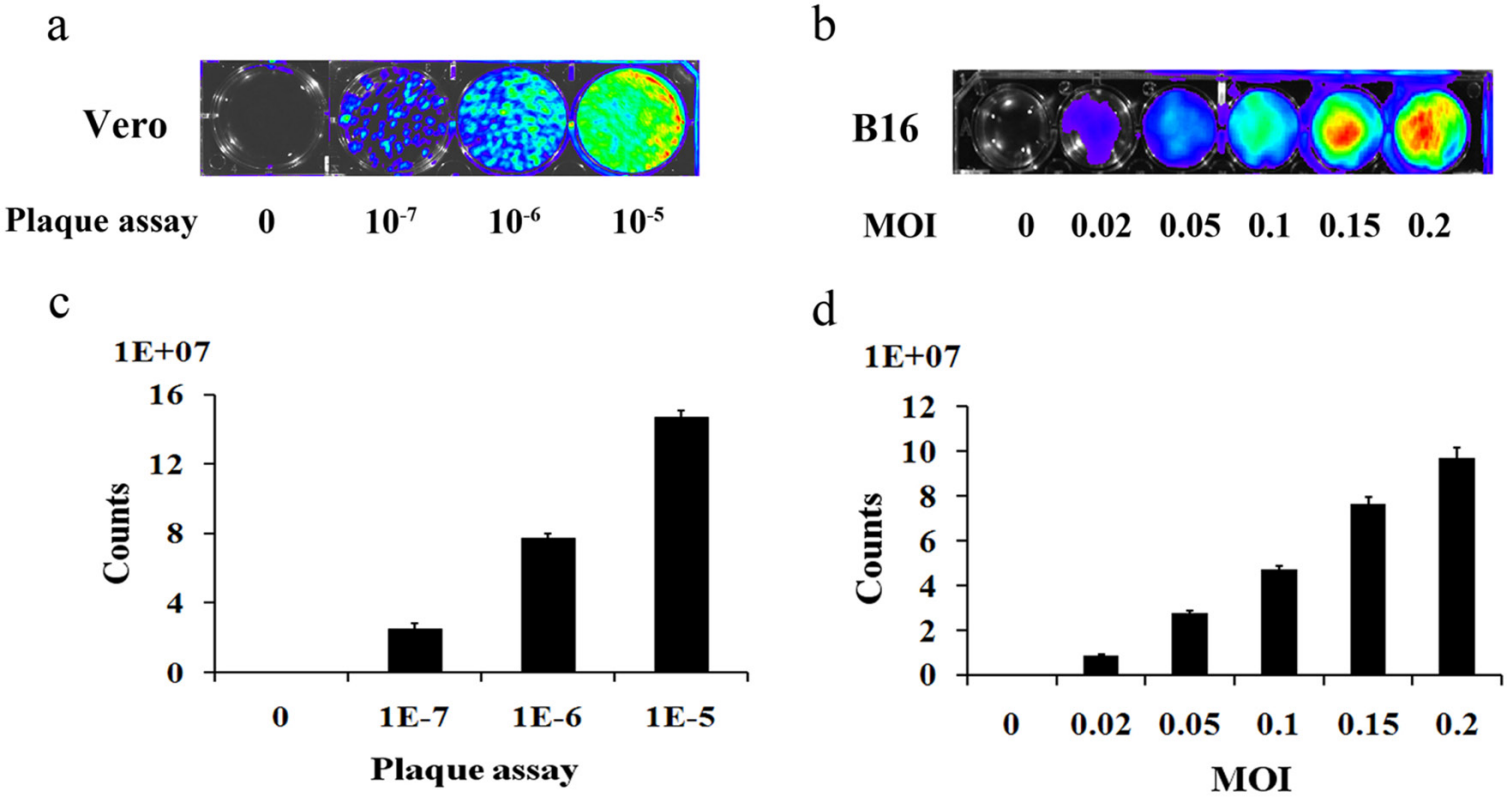
Plaque assay for the determination of the titer of purified vaccinia VG9-Luc **(a)** and the infection ability of vaccinia VG9-Luc on B16 cells **(b)** Purified vaccinia VG9-Luc was 10-fold diluted and infected to the Vero cells at the dilution of 10^-7^, 10^-6^ and 10^-5^ for 48 h. B16 cells were infected with increasing titers of VG9-Luc (0, 0.02, 0.05, 0.1, 0.15 or 0.2 MOI/well) for 48 h. Bioluminescence image was applied by addition of 150 μg/ml D-luciferin and 5 min incubation. Bar graph depicts the total photon counts of each well (means ± SD).

To determine whether vaccinia VG9-Luc had been successfully constructed and could infect tumor cells as expected, we infected the murine melanoma cell line B16 with VG9-Luc *in vitro*. First, we performed a plaque assay to determine the titer of the purified vaccinia VG9-Luc, and characterized the luciferase expression in a 6-well plate by luminescence imaging. As shown in Figure 1a and 1c, the firefly luciferase in vaccinia VG9-Luc was successfully expressed and the titer of the virus was as high as 10^8^ PFU/ml. We then infected B16 cells with increasing titers of vaccinia VG9-Luc. The luminescence imaging showed increasing luciferase activity in the cells infected with increasing titers of vaccinia VG9-Luc (Figure 1b and 1d). These data indicated that vaccinia VG9-Luc effectively infected tumor cells *in vitro*.

### Vaccinia VG9-Luc is capable of targeting tumor cells in nude mice

**Figure 2:**
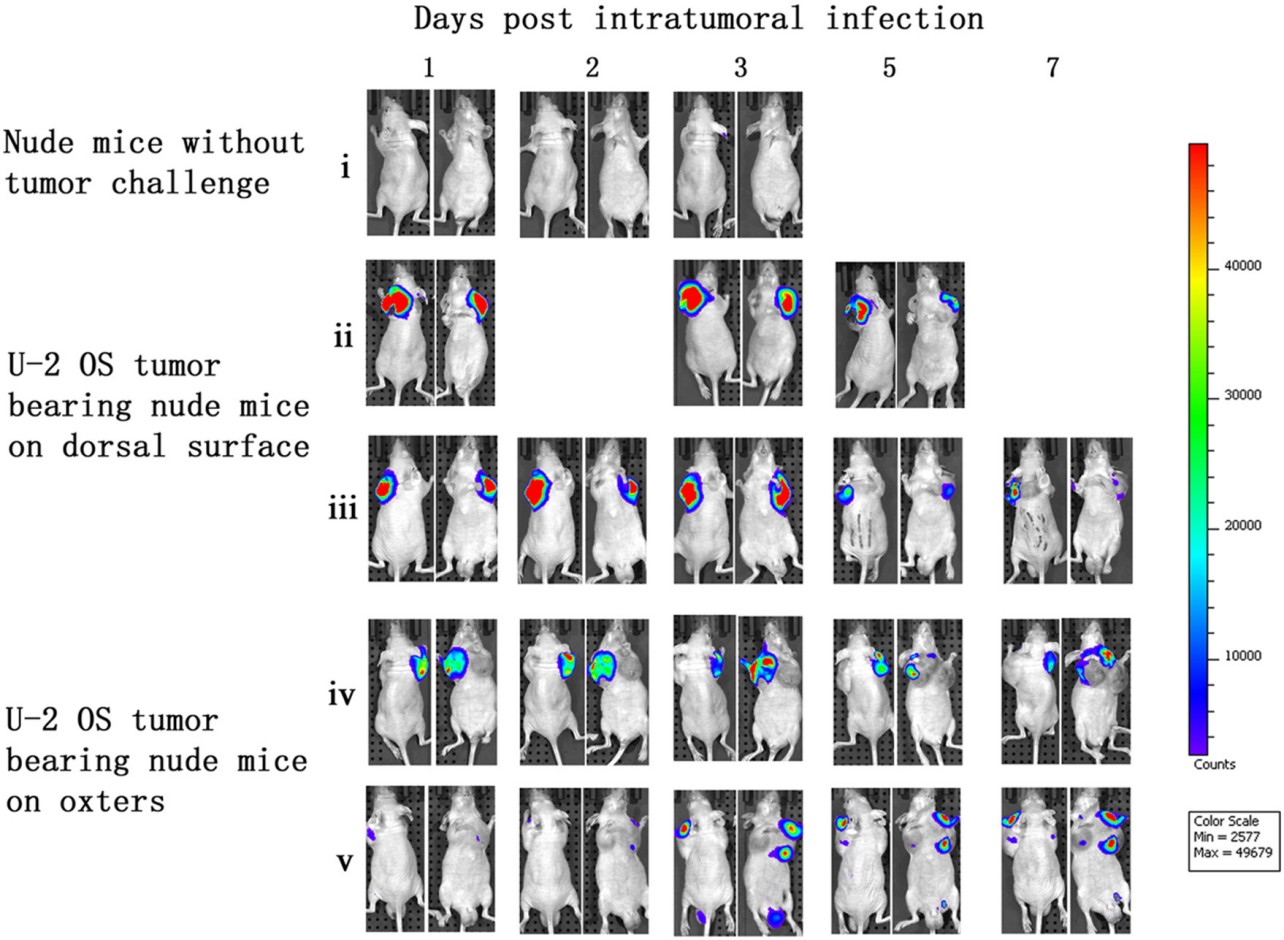
Characterization of luciferase activity in U-2 OS tumor-bearing nude mice intratumorally infected with VG9-Luc Nude mice were either challenged with U-2 OS cells or without tumor challenge. Two weeks after tumor inoculation, nude mice were intratumorally injected with 1×10 ^7^PFU of VG9-Luc. Luciferase activity was characterized by bioluminescence imaging on days 1, 2, 3, 5 and 7 after vaccinia infection. An integration time of 30 to 120 s with binning factors of 8 was used for bioluminescent image acquisition. **(i)** Bioluminescence image of subcutaneous injection of VG9-Luc into nude mice without tumor challenge. **(ii)** and **(iii)** Bioluminescence image of intratumoral infection of VG9-Luc into nude mice challenged with U-2 OS cells on dorsal surface. **(iv)** and **(v)** Bioluminescence image of intratumoral infection of VG9-Luc into nude mice challenged with U-2 OS cells on both oxters.

Immunocompromised nude mice bearing human osteosarcoma U-2 OS cells on their dorsal surfaces or in their oxters were used to investigate the tumor specificity of vaccinia VG9-Luc. Nude mice were infected with 1×10^7^PFU of VG9-Luc by intratumoral or intraperitoneal inoculation 2 weeks after tumor implantation. We performed bioluminescence imaging daily on all mice to monitor the progression of the infection and its tumor specificity. As shown in Figure 2, bioluminescence could not be detected in nude mice without tumor challenge, despite subcutaneous injection of vaccinia VG9-Luc on dorsal surface. However, considerable luciferase activity was detected locally after intratumoral infection with vaccinia VG9-Luc, regardless of the tumor position. Bioluminescence imaging over the course of infection showed strong photon flux on days 1, 2, and 3, which progressively decreased at days 5 and 7. The data indicated that subcutaneous injection of vaccinia virus in healthy tissue did not lead to viral replication and luciferase accumulation. Nevertheless, when the vaccinia virus was intratumorally injected, the virus quickly replicated and yielded strong luciferase activity at the exact position of the tumor, suggesting the tumor specificity of vaccinia VG9.

**Figure 3:**
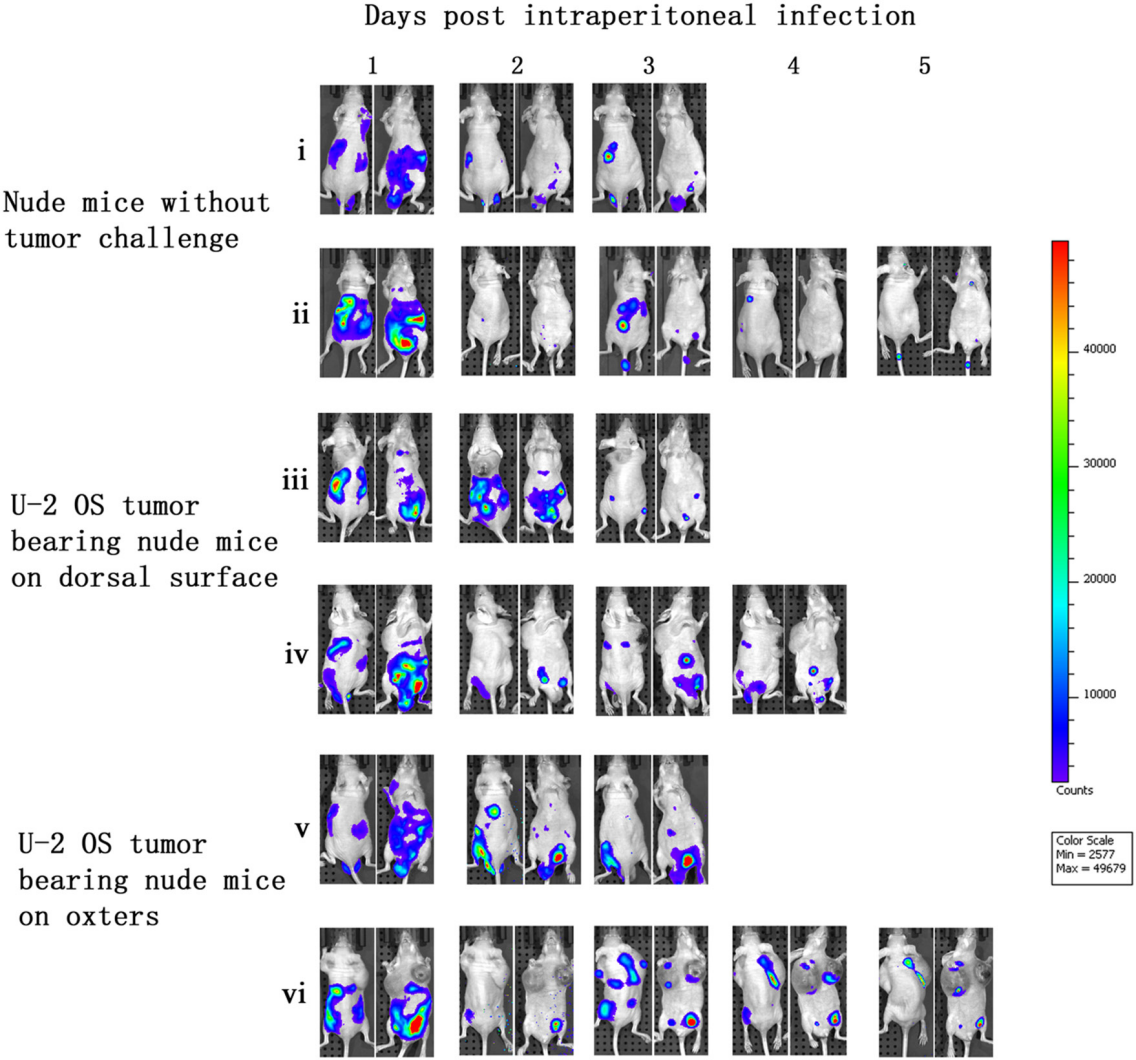
Characterization of luciferase activity in U-2 OS tumor-bearing nude mice intraperitoneally infected with VG9-Luc Nude mice were either challenged with U-2 OS cells or without tumor challenge. Two weeks after tumor inoculation, nude mice were intraperitoneally injected with 1×10^7^ PFU of VG9-Luc. Luciferase activity was characterized by bioluminescence imaging on days 1, 2, 3, 4 and 5 after vaccinia infection. An integration time of 30 to 120 s with binning factors of 8 was used for bioluminescent image acquisition. **(i)** and **(ii)** Bioluminescence image of intraperitoneal injection of VG9-Luc into nude mice without tumor challenge. **(iii)** and **(iv)** Bioluminescence image of intraperitoneal infection of VG9-Luc into nude mice challenged with U-2 OS cells on dorsal surface. **(v)** and **(vi)** Bioluminescence image of intraperitoneal infection of VG9-Luc into nude mice challenged with U-2 OS cells on both oxters.

After vaccinia VG9-Luc was intraperitoneally injected, its bioluminescence pattern was different than after intratumoral inoculation. As shown in Figure 3, the day after intraperitoneal infection with vaccinia VG9-Luc (day 1), the luciferase activity on the ventral and dorsal surface was high, in non- and -tumor-bearing nude mice. The bioluminescence gradually decreased over the course of infection. On days 4 and 5, luciferase activity in mice without tumor challenge could hardly be detected, but mice with U-2 OS tumor cells implanted in both oxters had luciferase activity at the tumor sites (Figure 3, vi). The data indicated that vaccinia VG9-Luc had the property of tumor selectivity, since the virus effectively targeted to the oxters from the abdominal cavity and accumulated in the tumor cells. However, mice bearing tumor cells on dorsal surface showed no bioluminescence in the tumor, suggesting that the virus had a longer route from the abdominal cavity to the back than to the oxters (Figure 2b, iv).

### Vaccinia VG9-Luc is capable of targeting tumor cells in C57BL/6 mice

**Figure 4:**
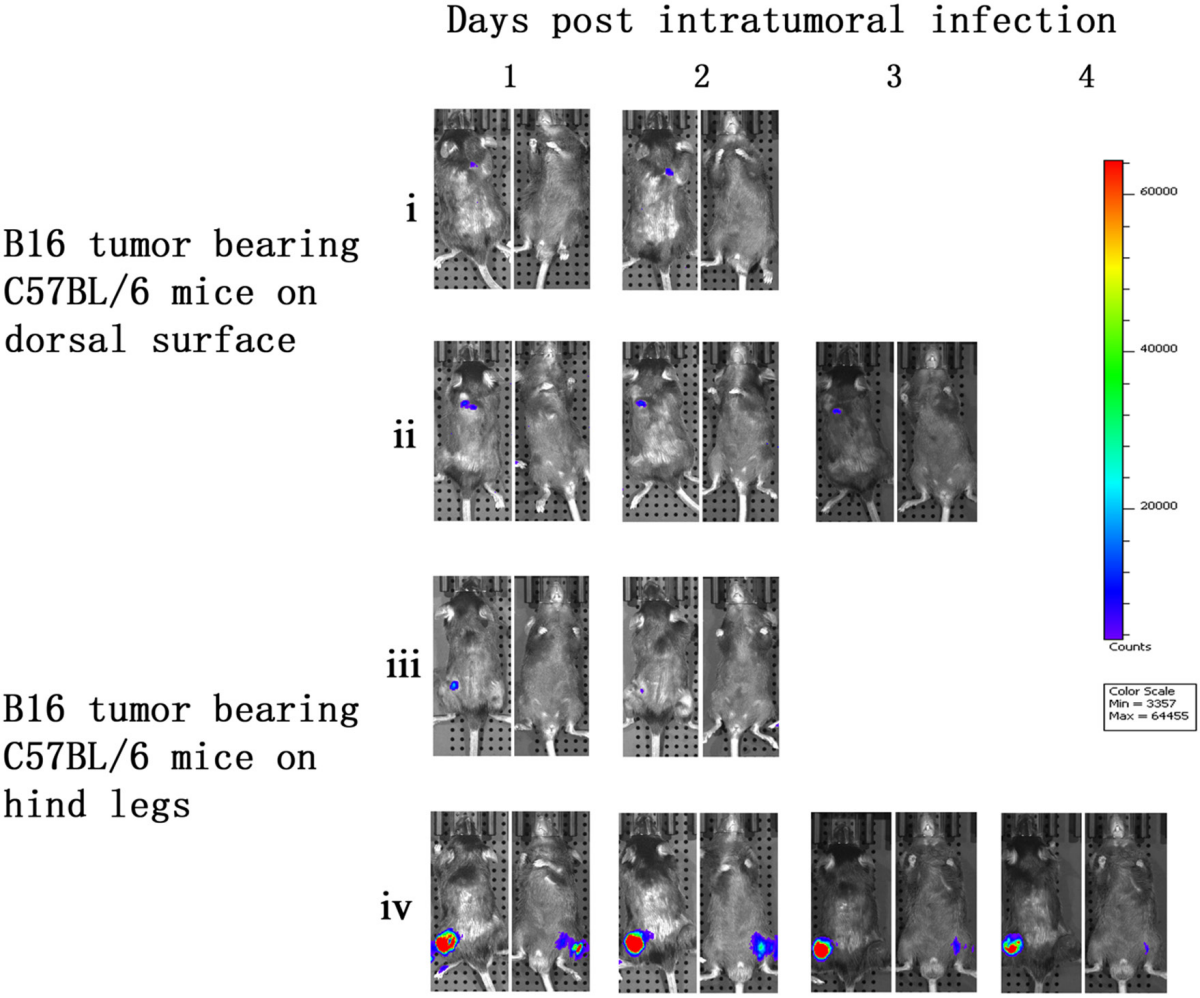
Characterization of luciferase activity in B16 tumor-bearing C57BL/6 mice intratumorally infected with VG9-Luc C57BL/6 mice were either challenged with B16 cells or without tumor challenge. One week after tumor inoculation, C57BL/6 mice were intratumorally injected with 1×10^7^ PFU of VG9-Luc. Luciferase activity was characterized by bioluminescence imaging on days 1, 2, 3 and 4 after vaccinia infection. An integration time of 30 to 120 s with binning factors of 8 was used for bioluminescent image acquisition. **(i)** and **(ii)** Bioluminescence image of intratumoral infection of VG9-Luc into C57BL/6 mice challenged with B16 cells on dorsal surface. **(iii)** and **(iv)** Bioluminescence image of intratumoral infection of VG9-Luc into C57BL/6 mice challenged with B16 cells on both hind legs.

To further demonstrate the tumor specificity of vaccinia VG9-Luc, immunocompetent C57BL/6 mice either challenged with B16 murine melanoma or not, were subsequently infected with 1×10^7^ PFU of VG9-Luc by intratumoral or intraperitoneal inoculation. As shown in Figure 4, C57BL/6 mice challenged with B16 tumors on their dorsal surfaces or hind legs had bioluminescence from luciferase activity after intratumoral infection. However, the photon flux in C57BL/6 mice was much lower than that in nude mice, except in mouse iv. The immune system in C57BL/6 mice may prompt the clearance of the vaccinia virus. The tumor size and the exact position of the viral inoculation may also affect the replication of the vaccinia virus.

**Figure 5:**
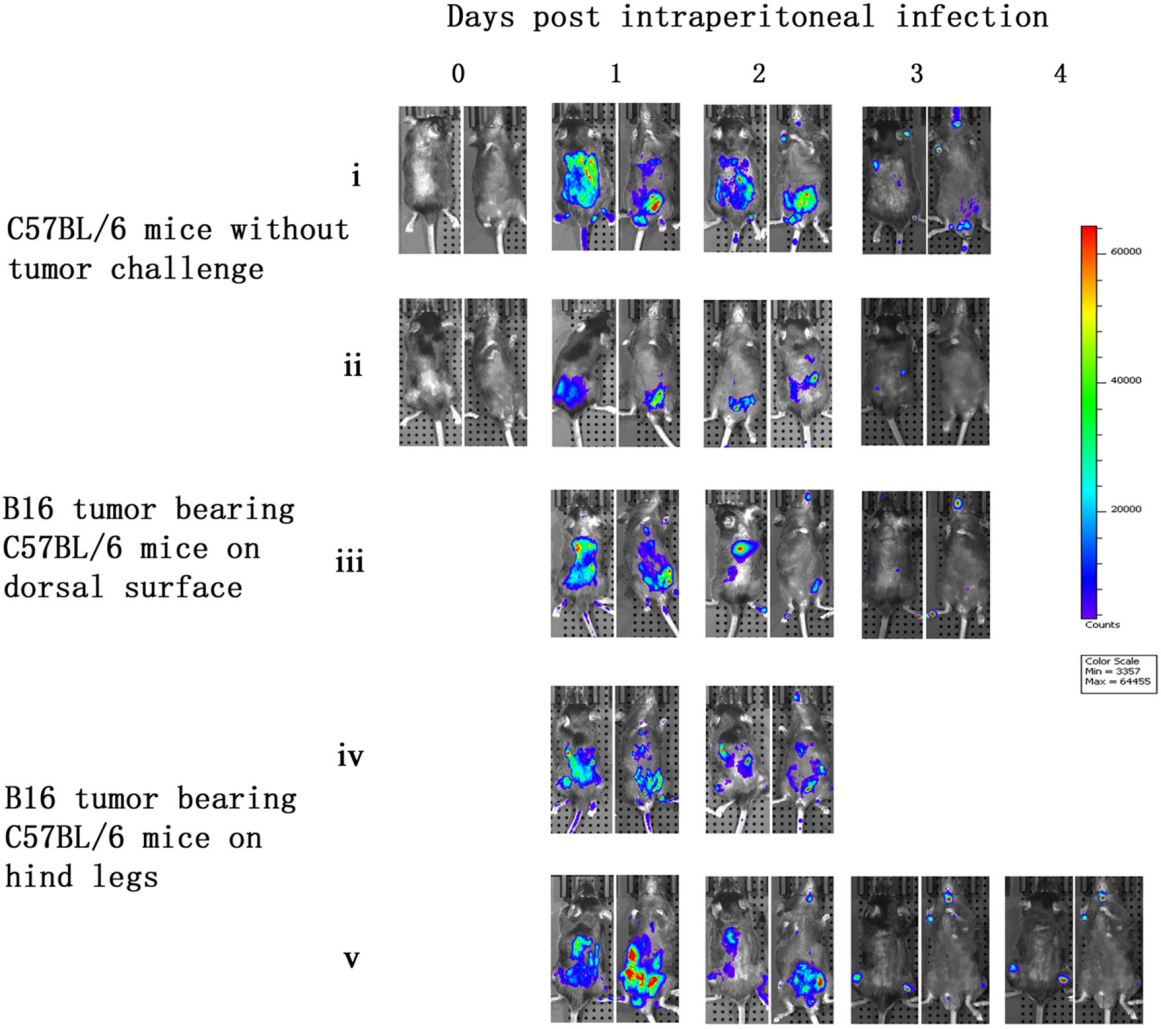
Characterization of luciferase activity in B16 tumor-bearing C57BL/6 mice intraperitoneally infected with VG9-Luc C57BL/6 mice were either challenged with B16 cells or without tumor challenge. One week after tumor inoculation, C57BL/6 mice were intraperitoneally injected with 1×10^7^ PFU of VG9-Luc. Luciferase activity was characterized by bioluminescence imaging on days 0, 1, 2, 3 and 4 after vaccinia infection. An integration time of 30 to 120 s with binning factors of 8 was used for bioluminescent image acquisition. **(i)** and **(ii)** Bioluminescence image of intraperitoneal injection of VG9-Luc into C57BL/6 mice without tumor challenge. **(iii)** Bioluminescence image of intraperitoneal infection of VG9-Luc into C57BL/6 mice challenged with B16 cells on dorsal surface. **(iv)** and **(v)** Bioluminescence image of intraperitoneal infection of VG9-Luc into C57BL/6 mice challenged with B16 cells on both hind legs.

C57BL/6 mice without tumor challenge were intraperitoneally injected with vaccinia VG9-Luc and bioluminescence imaging was performed immediately. No bioluminescence was detected on the day of infection (day 0). However, high luciferase activity was observed in the abdominal cavities of the C57BL/6 mice the day after infection (day 1). The luminescence dramatically decreased on day 2, and little or no luciferase expression was observed on day 3 (Figure 5). C57BL/6 mice that had received tumor challenge on the dorsal surface showed a similar result to mice that did not receive tumor challenge, and no vaccinia virus tumor selectivity was observed. Furthermore, C57BL/6 mice that had received tumor challenge on both hind legs had specific tumor targeting on days 3 and 4 after vaccinia VG9-Luc infection (Figure 5, v). The luminescence disappeared in the abdominal cavity and accumulated in both tumor legs. These results suggested that vaccinia virus had the ability to target tumor cells in immunocompetent mice. The lack of viral accumulation at the dorsal surface may be due to difficulty for vaccinia virus in the abdominal cavity to reach the dorsal surface, and the virus may have been cleared by the immune system before it reached the tumor position.

### Biodistribution of vaccinia VG9-Luc in C57BL/6 and nude mice

Normal organs including heart, liver, spleen, lung, kidney, brain, and blood, were harvested 2-7 d post-injection of vaccinia VG9-Luc, corresponding to the last day of bioluminescence imaging. Viral titers in the excised organs showed little or no plaques were formed from normal organs (Table 1), and luciferase activity was not detected in the tissue homogenates (data not shown).

**Table 1:** Biodistribution of vaccinia VG9-Luc in normal tissues

	Nude mice (PFU)	C57BL/6 (PFU)
blood	36 (0-74)	11 (0-15)
heart	0 (0-28)	0 (0-2)
Liver	0 (0-3)	0 (0-2)
Spleen	0 (0-12)	0 (0-15)
Lung	38 (0-121)	8 (0-22)
Kidney	0 (0-6)	0 (0-3)
brain	24 (0-60)	0 (0-2)

^a^Excised organs were homogenized in 1 ml of balanced salt solution. The virus was released by 3 cycles of freezing and thawing procedure. Plaque assays were applied to determine the quantity of virus remained in the organs.

In tumor tissues that received intratumoral injection of vaccinia VG9-Luc, the luciferase activity and viral titers were extremely high, in agreement with the results of the *in vivo* bioluminescence imaging (Table 2 and 3). The tumors that did not receive intratumoral injections of virus had no luciferase activity, although their viral titers were higher than those of the normal organs (over 10^3^ PFU/ml in nude mice or 10^2^ PFU/ml in C57BL/6). These results suggested that vaccinia virus not only replicated at the site of intratumoral injection, it also targeted to the tumors that did not receive the virus, although the low degree of infectivity was not detectable by luciferase activity assay.

**Table 2:** Luciferase activity biodistribution and plaque assays of vaccinia VG9-Luc in nude mice

				Luciferase activity	Plaque assay (PFU/ml)
Intraperitoneal Figure 3	Oxters	vi	left	791.3±171.5	69.5±18.1*10^5^
			right	1434.6±228.6	95.8±22.7*10^5^
		v	left	0	18.2±5.8*10^3^
			right	0	22.6±9.7*10^3^
	Dorsal surface	iii		0	35.4±8.6*10^3^
		iv		0	52.2±13.15*10^3^
Intratumoral Figure 2	Oxters	vi	left	0	27.8±6.7*10^3^
			right	8819.8±198.7	63.9 ±12.3*10^6^
		v	left	9799.3±467.7	78.3±16.15*10^6^
			right	1477.9±52.2	17.8±6.2*10^6^
	Dorsal surface	iii		10483.9±325.9	34.6±11.5*10^6^
		iv		1186.3±163.2	20.4±7.8*10^6^

^a^Nude mice were sacrificed on the last day of luminescene imaging. Tumors were harvested, homogenized in 1 ml of balanced salt solution. After 3 cycles of freezing and thawing procedure, the supernates were quantified by the Firefly Luciferase Reporter Gene Assay Kit for luciferase activity and by plaque assays for viral titers.

**Table 3:** Luciferase activity biodistribution and plaque assays of vaccinia VG9-Luc in C57BL/6 mice

		mouse		Luciferase activity	Plaque assay (PFU/ml)
Intraperitoneal Figure 5	Hind legs	iv	left	3550.2±54.8	7.3±3.15*10^4^
			right	1118.5±49.5	9.5±4.1*10^4^
		v	left	17203.6±247.1	5.2±3.3*10^6^
			right	33329.8±364.8	6.1±3.8*10^6^
	Dorsal surface	iii		0	37.5±8.7*10^2^
Intratumoral Figure 4	Hind legs	iv	left	33957.3±91.0	13.6±5.15*10^6^
			right	0	14.4±6.8*10^2^
		iii	left	0	14.8±5.9*10^2^
			right	0	26.2±13.4
	Dorsal surface	i		0	34.3±6.7*10^2^
		ii		0	22.9±7.1*10^2^

^a^C57BL/6 mice were sacrificed on the last day of luminescene imaging. Tumors were harvested, homogenized in 1 ml of balanced salt solution. After 3 cycles of freezing and thawing procedure, the supernates were quantified by the Firefly Luciferase Reporter Gene Assay Kit for luciferase activity and by plaque assays for viral titers.

In addition, the tumor tissues in nude mice or C57BL/6 mice that received intraperitoneal injection of vaccinia VG9-Luc showed high luciferase activity and viral titers (Table 2 and 3), suggesting effective tumor targeting by vaccinia virus. Moreover, the viral titers in the dorsal surface were much lower than in the hind legs or oxters, indicating the tumors in the dorsal surface were more difficult for the virus to reach, perhaps due to the long distance to the dorsal surface from the abdominal cavity. The luciferase activity in the tumor homogenates corresponded exactly with the results of the bioluminescence imaging, which showed the direct correlation between the luciferase assays *in vivo* and *in vitro*.

## DISCUSSION

Tumor selectivity is a natural property of vaccinia virus, and the deletion of the gene encoding TK further enhances the tumor targeting ability of vaccinia virus. Owing to limited technology, the evaluation of tumor selectivity previously depended mainly on comparisons of viral titers from excised normal tissues and tumors using plaque assays. A novel imaging modality for living animals, *in vivo* bioluminescence imaging, has received considerable attention in recent years. *in vivo* bioluminescence imaging uses the luciferase family of proteins to create signature bioluminescent outputs that are captured by advanced cameras. Luciferases operate in tandem with their luciferin substrates to generate light via an oxidative decarboxylation reaction that forms an excited state intermediate that releases energy in the form of photons as it returns to its ground state [20]. To exploit the advantages of live imaging for studies of tumor selectivity, we generated the recombinant vaccinia Tian Tan strain VG9 that expresses firefly luciferase to enable bioluminescence imaging of vaccinia virus infection in living mice.

We established 2 animal models for the evaluation of tumor selectivity: immunocompromised nude mice bearing human osteosarcoma U-2 OS cells and immunocompetent C57BL/6 mice challenged with B16 murine melanoma cells. We found that VG9 with TK deletion specifically targeted to tumors in both immunocompromised and immunocompetent mice. After intratumoral infection, vaccinia VG9 effectively replicated in tumors and high luciferase activity was detected at the local site of the tumor. Nevertheless, no luciferase activity was detected after subcutaneous injection of vaccinia VG9 on the dorsal surface, suggesting that vaccinia virus could not proliferate in normal tissues. Furthermore, vaccinia virus showed tumor selectivity after systemic administration. After intraperitoneal infection with vaccinia VG9, bioluminescence image revealed viral accumulation in tumor positions in nude mice bearing U-2 OS tumors on the oxters and C57BL/6 mice bearing B16 tumors on the hind legs. Unfortunately, neither nude mice nor C57BL/6 mice bearing tumors on the dorsal surface had luciferase activity at the tumor sites. However, plaque assays revealed that, although the tumors showed no bioluminescence, their viral titers were higher than those of normal tissues, which suggested that the virus also accumulated in these tumors, albeit at a low and undetectable level. The differences in bioluminescence at the dorsal surface and the oxters/hind legs may be due to their distances from the abdominal cavity. After intraperitoneal injection, the virus accumulated in the abdominal cavity and reached the oxters or hind legs. The dorsal surface is relatively farther away for the virus, which may be cleared by the immune system before it gets to the tumors. This phenomenon indicated that both the tumor position and the route of administration affect the tumor targeting ability of vaccinia virus. It is also suggested that changing the route of administration (e.g. intravenous injection) would lead to different results.

Vaccinia virus is a heterologous organism that induces the host immune response. One potential limitation of using vaccinia virus as an antitumor agent is the rapid antiviral immune response and subsequent virus clearance, which limit the use of vaccinia virus in immunocompetent hosts [21]. Thus, we compared the effects of the immune response by inoculating immunocompromised nude mice and immunocompetent C57BL/6 mice. As expected, the nude mice had higher bioluminescent intensity for a longer duration than C57BL/6 mice, after both intratumoral and intraperitoneal administration. In addition, plaque assays revealed that the PFU of the tumors from nude mice were an order of magnitude higher than the PFU of the tumors from C57BL/6 mice, indicating that the immune response in C57BL/6 mice played a role in the clearance of the exogenous vaccinia virus.

In summary, we constructed a TK deletion vaccinia Tian Tan strain VG9 and evaluated its tumor selectivity by *in vivo* bioluminescence imaging. It is encouraging that vaccinia VG9 displayed tumor-specific targeting after systemic administration of intraperitoneal. These data provide evidence for the safety and efficacy of the clinical application of vaccinia virus, which could be a promising approach for cancer therapy. As a next step, we will evaluate the tumor selectivity of vaccinia VG9 after systemic administration by intravenous injection.

## MATERIALS AND METHODS

### Cell lines

Cell lines, including B16 (murine melanoma), U-2 OS (human osteosarcoma), HEK-293 (human embryonic kidney cells), and Vero and BSC-40 (both African green monkey kidney epithelial cells) were cultured in DMEM or RPMI 1640 with 10% heat-inactivated fetal bovine serum and 0.1% penicillin-streptomycin in a 5% CO_2_ incubator at 37°C. B16 and U-2 OS were inoculated into C57BL/6 mice or BALB/C nu/nu (nude) mice. HEK-293 cells were used as an intermediate for vaccinia virus transfection. Vero and BSC-40 were used for *in vitro* viral replication and plaque assays.

### Animals

C57BL/6 and nude mice were acquired from Cavens Laboratory Animal (Changzhou, China). All animals were maintained under specific pathogen-free conditions; all procedures were performed in accordance with the Laboratory animal-Guideline of welfare ethical review of Chinese Institutional Animal Care and Use Committee (IACUC).

### Vaccinia

The VG9 strain carrying Luc (VG9-Luc) was generated using a previously described protocol [22]. VG9 was obtained from the National Institutes for Food and Drug Control (NIFDC, Beijing, China). The vaccinia shuttle plasmid (pCB) used for the generation of the recombinant vaccinia virus was provided by Professor Xin Yuan Liu (Institute of Biochemistry and Cell Biology, Shanghai Institutes for Biological Sciences, The Graduate School, Chinese Academy of Sciences). The pCB plasmid contains the flanking sequences of TK, which facilitated homologous recombination into the whole TK gene. The Luc DNA was obtained from the pGL3-Basic plasmid (Promega, Madison, Wisconsin, USA) and placed under the control of the vaccinia synthetic early/late promoter of pCB.

The recombinant pCB-Luc plasmid was transfected into HEK-293 cells with Effectene^®^ Transfection Reagent (Qiagen, Venlo, Netherlands) 2 h after infection with wild-type VG9, so that the Luc gene with TK flanking sequences could replace the TK gene of the VG9 strain by homologous recombination. Recombinants were then selected in Vero cells in the presence of mycophenolic acid, xanthine, and hypoxanthine as xanthine-guanine phosphoribosyltransferase (XGPRT) selection. The VG9-Luc virus was plaqued for at least 6 rounds of selection to confirm that a pure recombinant stock was obtained. Vaccinia virus was propagated in BSC-40 cells and purified by centrifugation through a sucrose gradient. The titer of purified vaccinia VG9-Luc was determined by plaque assay on Vero cells and expressed as plaque-forming units (PFU)/ml.

### Expression of luciferase

BSC-40 cells seeded in 6-well plates were infected with a 0.1 multiplicity of infection (MOI, or PFU/cell) of VG9-Luc in 2% fetal bovine serum for 2 h, then the infection medium was removed and replaced with fresh growth medium. After 48 h of incubation, cells and supernatants were harvested. Following 3 freeze-thaw cycles, luciferase activity was quantified with a Firefly Luciferase Reporter Gene Assay Kit (Beyotime, Shanghai, China) and a SpectraMax^®^ M5 Multi-Mode Microplate Reader (Molecular Devices, CA, USA).

*In vitro* infection of tumor cells by vaccinia VG9-Luc B16 cells were plated at 2×10^5^ cells/well in 24-well plates and grown overnight. The cells were infected with VG9-Luc at different MOIs (0, 0.02, 0.05, 0.10, 0.15, and 0.20). 48 h after infection, bioluminescence was quantified by adding 150 μg/ml D-luciferin (Promega) to culture medium 5 min before imaging. An integration time of 10s was used for luminescent image acquisition with the IVIS system (PerkinElmer, CA, USA).

### Tumor models

To generate human osteosarcoma tumor models, the immunocompromised nude mice were subcutaneously implanted with 2×10^6^ U-2 OS human osteosarcoma cells on the dorsal surface or both the left and right oxters. After 10 d of tumor growth, tumor volumes were monitored with a digital caliper. Tumor volume (mm^3^) was estimated by the formula (L×H×W)/2, where L is the length, W is the width, and H is the height of the tumor in millimeters. Similarly, the immunocompetent C57BL/6 mice bearing subcutaneous melanoma tumors were used 1 week after subcutaneous implantation of 1×10^6^ B16 murine melanoma cells on the dorsal surface or both the left and right hind legs. Mice were 4-6 weeks old for all studies. The numbers of male and female mice in various experimental groups were matched, although we did not observe a difference between the sexes in susceptibility to vaccinia infection. When tumors reached 50-300 mm^3^, 1×10^7^ PFU of purified VG9-Luc, suspended in 100 μl of phosphate-buffered saline (PBS), were injected intraperitoneally or intratumorally into mice. Beginning the day after vaccinia virus inoculation, animals were imaged daily for bioluminescence detection.

### *In vivo* bioluminescence imaging

*In vivo* bioluminescence imaging was performed using a cooled CCD camera system (IVIS Imaging System, PerkinElmer). Before imaging, 15 mg/mL D-luciferin (potassium salt; Promega) in normal saline was intraperitoneally injected at a dose of 150 mg/kg body weight. Mice were placed in the light-tight chamber of the CCD camera system and 2% isoflurane anesthesia in air was delivered via a nose cone system. Mice were imaged daily from day 1 after vaccinia VG9-Luc infection. An integration time of 30 to 120 s, with binning factors of 8, was used for luminescent image acquisition. The signal intensity was quantified as the flux of all detected photon counts within a region of interest (ROI) prescribed over the ventral or dorsal torso using the LivingImage software package.

### Virus titration from infected organs

Organs including heart, liver, spleen, lung, kidney, brain, and tumors, were removed on the day of death, homogenized in 1 ml of balanced salt solution, and immediately stored at −80°C. Whole blood was obtained by cardiac puncture and diluted in 1 ml of medium with 2% fetal bovine serum. Tissue homogenates and blood were lysed by 3 cycles of freezing and thawing, and centrifuged for 5 min at 3000 ×*g* at 4°C. Supernatants were aliquoted and virus titers were determined by plaque assay on BSC-40 cells. Meanwhile, the luciferase activity of the organ solutions was quantified with the Firefly Luciferase Reporter Gene Assay Kit (Beyotime).

### Statistical analysis

Data are presented as mean ± standard deviation. Statistical testing was performed by analysis of variance (ANOVA) using SPSS 19.0 software.

## Abbreviations

VTT: vaccinia virus Tian Tan strain
VG9: vaccinia virus Guang9 strain
TK: thymidine kinase
Luc: firefly luciferase
VG9-Luc: VG9 strain carrying Luc
PFU: plaque-forming unit
MOI: multiplicity of infection
